# Keeping Balance on a Moving Platform: A Qualitative Interview Study of Young Adults Living with Epilepsy and Implications for Neurological Nursing

**DOI:** 10.3390/nursrep16090329

**Published:** 2026-09-09

**Authors:** Janet Froulund Jensen, Liv Gølnitz Hjorhöy, Belle Mia Ingerslev Loft

**Affiliations:** 1 Department of Regional Health Research, University of Southern Denmark, 5230 Odense, Denmark; 2 Department of Neurology, Zealand University Hospital, 4000 Roskilde, Denmark; lhju@regionsjaelland.dk; 3 Department of Health Strategic Planning, Region Zealand, 4140 Sorø, Denmark; 4 Department of Neurology, Copenhagen University Hospital Rigshospitalet, 2600 Glostrup, Denmark; belle.mia.ingerslev.loft@regionh.dk; 5 Department of Clinical Medicine, University of Copenhagen, 2200 Copenhagen, Denmark

**Keywords:** epilepsy, young adults, qualitative research, self-management, person-centered care, neurological nursing, reflexive thematic analysis, developmental transitions

## Abstract

**Background/Objectives:** Supporting everyday self-management is a central component of person-centered neurological nursing for young adults living with epilepsy. However, little is known about how young adults experience and negotiate everyday life with epilepsy during a developmental stage characterized by increasing independence and major life transitions. This study aimed to explore how young adults experience and negotiate everyday life with epilepsy. **Methods:** A qualitative exploratory study informed by a hermeneutic approach was conducted to explore participants’ experiences. Twelve young adults aged 18–29 years participated in individual virtual interviews. Interviews followed a semi-structured guide and were analyzed using reflexive thematic analysis to identify patterns across participants’ experiences. The study was reported according to the Standards for Reporting Qualitative Research (SRQR). **Results:** The analysis identified a main theme, Keeping balance on a moving platform, which captured how young adults experienced living with epilepsy as a continuous balancing act within an unpredictable condition. Participants described continuously balancing independence with vulnerability while managing seizures, cognitive challenges, and concerns related to safety and social participation. Familiar digital and non-digital tools supported everyday self-management by helping participants structure daily routines, compensate for cognitive difficulties, and maintain a sense of normality. **Conclusions:** Living with epilepsy in young adulthood requires continuous adaptation to cognitive, emotional, and practical challenges that extend beyond seizure control. This study extends current knowledge by showing that self-management involves continuous negotiation between independence, vulnerability, and everyday participation rather than symptom control alone. The findings provide insights into person-centered neurological nursing by highlighting the importance of individualized support for managing cognitive challenges, everyday self-management, and transitions into education, employment, and family life.

## 1. Introduction

Epilepsy is a chronic neurological condition that influences physical, psychological, and social aspects of daily living across the lifespan [1]. Many individuals develop epilepsy in childhood or adolescence and continue to live with the condition into young adulthood, a period marked by increasing independence and major transitions related to education, work, relationships, and identity formation [2,3]. During young adulthood, individuals often navigate uncertainty related to seizure occurrence, cognitive difficulties, medication adherence, and social exposure. This developmental period is not merely chronological but characterized by increasing independence, identity exploration, and movement towards adult roles [3]. For individuals living with a chronic illness from childhood, this transition may be shaped by ongoing illness management and increased vulnerability, while social participation and appearing “normal” remain highly relevant concerns [4].

Unpredictable seizures remain a significant clinical challenge, with approximately 30% of individuals continuing to experience seizures despite treatment, commonly referred to as drug-resistant epilepsy [5]. This persistent unpredictability contributes to psychological distress and social isolation among people with epilepsy [6,7,8] and is further associated with broader psychosocial challenges and reduced quality of life [9]. Seizure unpredictability may further undermine perceived control, disrupt daily routines, and complicate decisions regarding education, employment, and social participation [10,11,12,13]. These challenges can negatively affect well-being, autonomy, and confidence in everyday self-management [13], particularly during a developmental phase characterized by increasing expectations of independence and social involvement [4].

Research has described epilepsy as a condition that extends beyond seizure control, covering psychosocial and everyday life challenges [10,14,15]. Qualitative research shows that living with epilepsy involves managing unpredictability, stigma, and disruptions to daily routines and social involvement [15]. These challenges may intensify during young adulthood, a period characterized by transitions towards independence, education, and relationships [2,3,16]. Despite increasing attention to self-management, there remains limited knowledge about how young adults themselves experience and manage epilepsy in everyday life, and how support can be tailored to their needs [17]. Existing epilepsy research has largely focused on seizure control, treatment adherence, or mixed-age adult populations [18], while less is known about the experiences of young adults navigating developmental transitions alongside chronic illness. Greater understanding of these experiences may help neurological nurses provide person-centered support that reflects young adults’ everyday self-management needs and developmental context. Therefore, this study aimed to explore how young adults experience and negotiate everyday life with epilepsy.

## 2. Materials and Methods

### 2.1. Design

A qualitative study using reflexive thematic analysis, as described by Braun and Clarke, 2021 [19], was conducted to explore patterns in how young adults understand and negotiate living with epilepsy in everyday life. Reflexive thematic analysis was chosen because it provides a flexible yet rigorous approach for identifying and interpreting patterns of shared meaning across qualitative data [19]. The study was informed by a hermeneutic approach [20], emphasizing interpretation as an iterative and reflexive process shaped by the researchers’ engagement with the data. This approach aligns with reflexive thematic analysis, in which themes are developed through active interpretation rather than discovered within the data [19]. Epilepsy was conceptualized as a neurological condition with a biological basis, while participants’ experiences were understood as contextually shaped processes of meaning-making influenced by social, relational, and developmental factors [19]. Within this framework, themes were interpreted as patterns of shared meaning generated through the researchers’ active engagement with the data, rather than as objective or essential features of experience [19]. Individual semi-structured interviews were used to generate in-depth descriptions of how epilepsy influenced daily routines, relationships, decisions about whether to disclose the condition, and strategies for managing seizure unpredictability [21]. Digital and non-digital tools were explored not as interventions in themselves, but as part of how young adults negotiate everyday life with an unpredictable condition. The study was reported in accordance with the Standards for Reporting Qualitative Research (SRQR) [22].

### 2.2. Participant and Setting

Young adults aged 18–30 years with lived experience of epilepsy were invited to participate. This age range was selected to reflect young adulthood as a developmental phase characterized by increasing independence and movement towards adult roles [3]. Recruitment took place between June–July 2021 via the Epilepsy Association’s website and a Facebook page targeting young adults with epilepsy. The Facebook post resulted in the inclusion of participants who were not affiliated with the Epilepsy Association. Additionally, some participants were not Facebook users themselves but had received the study invitation through relatives who had encountered the post on the platform. Inclusion criteria were: (1) age 18–30 years and (2) experience of managing epilepsy in everyday life. Eligibility was assessed by the author, LGH, during the initial contact with potential participants based on age, self-reported diagnosis of epilepsy, and experience of managing epilepsy in everyday life. Epilepsy diagnoses were not independently verified through medical records. No restrictions were placed on seizure type, duration of epilepsy, or disease management strategies, as variation was considered important for capturing diverse perspectives.

Participants were recruited on an ongoing basis as eligible individuals responded to the study invitation. Twelve participants were enrolled and completed individual virtual interviews; none subsequently declined or withdrew. Recruitment and interviewing proceeded concurrently and were discontinued after 12 interviews. The sample size was considered sufficient in relation to the study aim, the specificity of the sample, and the depth of the data generated, in line with the concept of information power [23]. Analytic sufficiency was therefore considered in relation to information power rather than theme saturation. The sample included individuals from all five regions of Denmark, with one participant residing abroad. Although the eligible age range was 18–30 years, the recruited participants were aged 18–29 years (median = 25.5). Participants underwent diagnostic evaluation and received treatment follow-up across neurology departments and outpatient clinics, a specialized epilepsy hospital, and general practice. All participants reported daily use of antiseizure medication, ranging from one to three medications. Five participants reported one or more comorbid conditions, including neurological, musculoskeletal, psychological, autoimmune, allergic, and migraine-related conditions. Nine participants reported cognitive difficulties. Psychological comorbidities included depression and a stress-related disorder. Participants did not report any neurodevelopmental conditions. However, nine participants reported cognitive problems, including concentration difficulties (*n* = 7), impaired memory (*n* = 8), learning difficulties (*n* = 3), and other cognitive difficulties related to executive functioning and sense of time (*n* = 1). One participant reported a diagnosis of juvenile myoclonic epilepsy; the remaining participants were unable to report their specific type of epilepsy. Participant characteristics are presented in Table 1.

### 2.3. Data Collection

Interviews were conducted between June and July 2021 via Zoom (online platform) by the second author [24]. Most participants participated from their homes, while one participant was staying at a holiday home. All participants were alone during the interviews; in some cases, family members were present elsewhere in the residence but did not participate. Participants received verbal and written information and provided written informed consent before the interview. A semi-structured interview guide was developed to support open-ended exploration of participants’ experiences of living with epilepsy. Topics covered include bodily experiences, social relationships, everyday environments, temporality, and supportive technologies Table 2. The guide provided a flexible framework to support rich, in-depth experiences, which were subsequently analyzed using reflexive thematic analysis [19]. Probes and clarifying questions (e.g., “*What happened then?*” and “*Can you elaborate on …?*”) were used to elicit a more detailed picture. Interviews lasted 30–80 min and were audio-recorded and transcribed verbatim. A random sample of the transcripts were checked against the audio recordings to verify transcription accuracy. Only audio was recorded; no video was recorded. Audio recordings and transcripts were stored on a secure, access-restricted drive. Only participants’ first names were collected as direct identifiers. Data were pseudonymized using participant codes (P1–P12), which were also used when presenting quotations in the manuscript. The interviews, transcriptions, and analysis were conducted in Danish. Participant quotations were translated from Danish into English as closely as possible to the participants’ original wording and spoken expression. Minor linguistic adjustments were made where necessary to ensure appropriate English wording and comprehension while preserving the original meaning.

### 2.4. Data Analysis

Data were analyzed using reflexive thematic analysis guided by the six-phase approach outlined by Braun and Clarke [19] as an iterative rather than linear process. First, all transcripts were read repeatedly to achieve familiarity with the data and to gain an overall sense of the dataset. Second, initial codes were generated across the entire dataset, focusing on features relevant to participants’ descriptions and negotiations of seizure unpredictability and everyday life. Coding was conducted inductively and iteratively, while remaining attentive to the research aim. JFJ and LGH conducted the initial analysis, including the development of initial themes and coding of the transcripts. Third, codes were collated into potential themes representing patterns of shared meaning. Fourth, themes were reviewed and refined in relation to both coded extracts and the dataset as a whole to ensure coherence and distinctiveness. Fifth, the developing analysis was discussed within the full research team, and themes were refined collaboratively by all three investigators. Variation in participants’ experiences was considered throughout theme development, including perspectives that differed from the predominant patterns [23]. Such variation was retained within the analysis to reflect the range and complexity of participants’ experiences [25,26]. During this process, a central organizing concept (i.e., main theme) was identified that captured patterns across the dataset. This main theme linked and contextualized themes and sub-themes, providing an interpretative framework for understanding how young adults negotiated living with epilepsy. Themes were conceptualized as patterns of shared meaning underpinned by the main theme rather than as summaries of topics or categories. This analytical approach supported an understanding of shared patterns of meaning while preserving variation in individual experiences, thereby providing insights relevant to person-centered neurological nursing care. The hermeneutic orientation informed the analysis through a continuous movement between the whole and the parts and back to the whole, reflecting the hermeneutic circle [20]. Initial codes and developing themes were interpreted in relation to participants’ broader experiences and repeatedly reconsidered as the researchers’ understanding of the data developed. Through this iterative interpretative process, the researchers’ pre-understandings were challenged and revised in dialogue with the data, allowing for a fusion of horizons and the development of new understandings [20]. In line with reflexive thematic analysis, themes were understood as actively constructed through the researchers’ analytic engagement with the data rather than as entities emerging independently from the dataset [19]. Ongoing reflexive dialogue within the research team supported the critical examination of analytic decisions, interpretations, and assumptions throughout the analytical process. Table 3 shows the analytical process.

### 2.5. Reflexivity

The research team consisted of three nurse investigators with backgrounds in nursing and health science. JFJ and BMIL were senior nursing researchers with clinical and research experience in neurology and qualitative research, working in two different Danish neurological departments. LGH was a clinical nurse specialist in neurology with less experience in research than JFJ and BMIL. The study provided LGH with an opportunity to gain research experience and explore an interest in further research training. At the beginning of each interview, LGH introduced herself as a nurse with limited knowledge of epilepsy and its treatment, emphasizing that participants were the experts on their own experiences. This positioning encouraged participants to describe their experiences without assuming shared clinical understanding. JFJ and LGH were affiliated with the Department of Neurology at Zealand University Hospital, while BMIL was affiliated with the Department of Neurology at Copenhagen University Hospital. None of the investigators were involved in direct clinical care of the participants. Participants were informed of LGH’s professional background as a clinical nurse specialist in neurology and of the purpose of the study before the interview. The researchers acknowledged that their clinical and academic backgrounds informed their understanding of epilepsy and neurological conditions. This pre-understanding provided clinical sensitivity to participants’ experiences but also had the potential to shape which aspects of the data were emphasized and how they were interpreted. Rather than attempting to set aside this pre-understanding, it was recognized as part of the interpretative process and critically examined throughout the analysis [20]. Differences in interpretations within the research team were discussed through ongoing reflexive dialogue and engagement with the data, allowing individual assumptions and interpretations to be critically examined throughout the analytic process [19,20]. Trustworthiness was supported through collaborative theme development and iterative discussions within the research team [19,25,26]. Participants were not asked to validate the interview transcripts or the subsequent analysis. Consistent with the interpretive and reflexive approach, themes were understood as interpretations developed through the researchers’ engagement with the data rather than as findings requiring confirmation by participants [19].

### 2.6. Ethical Considerations

Participants received written and verbal information about the study, confidentiality, and their right to withdraw at any time without consequences. Written informed consent was obtained from all participants. The study was registered with the Data and Development Support Unit, Region Zealand (REG-066-2021), with data-protection approval obtained on 11 June 2021, and conducted in accordance with the Declaration of Helsinki and national ethical guidelines for nursing research [27]. According to Danish legislation, ethical approval from a Research Ethics Committee is not required for interview studies that do not involve biological material or healthcare interventions.

## 3. Results

### 3.1. Main Theme: Keeping Balance on a Moving Platform

The main theme, “Keeping balance on a moving platform”, illustrated the balancing act of living with an unpredictable condition. Across the dataset, participants described living with epilepsy as a continuous effort to maintain equilibrium in the context of unpredictability. Daily life involved ongoing adjustments as they navigated seizures, cognitive difficulties, and concerns related to safety and social exposure. Routines, support, and personal strategies contributed to a sense of stability; however, unexpected symptoms or changes in bodily or cognitive functioning could quickly disrupt this balance. Epilepsy influenced not only physical functioning but also daily rhythms, relationships, and self-perception. Participants actively sought to create structure and predictability, while remaining aware that their condition could interrupt plans at any moment. Their experiences reflected a recurring pattern of constant negotiation between managing vulnerability and sustaining a sense of normality. The balancing act was particularly evident in situations where participants navigated tensions between independence and safety in everyday life.

**Figure 1:**
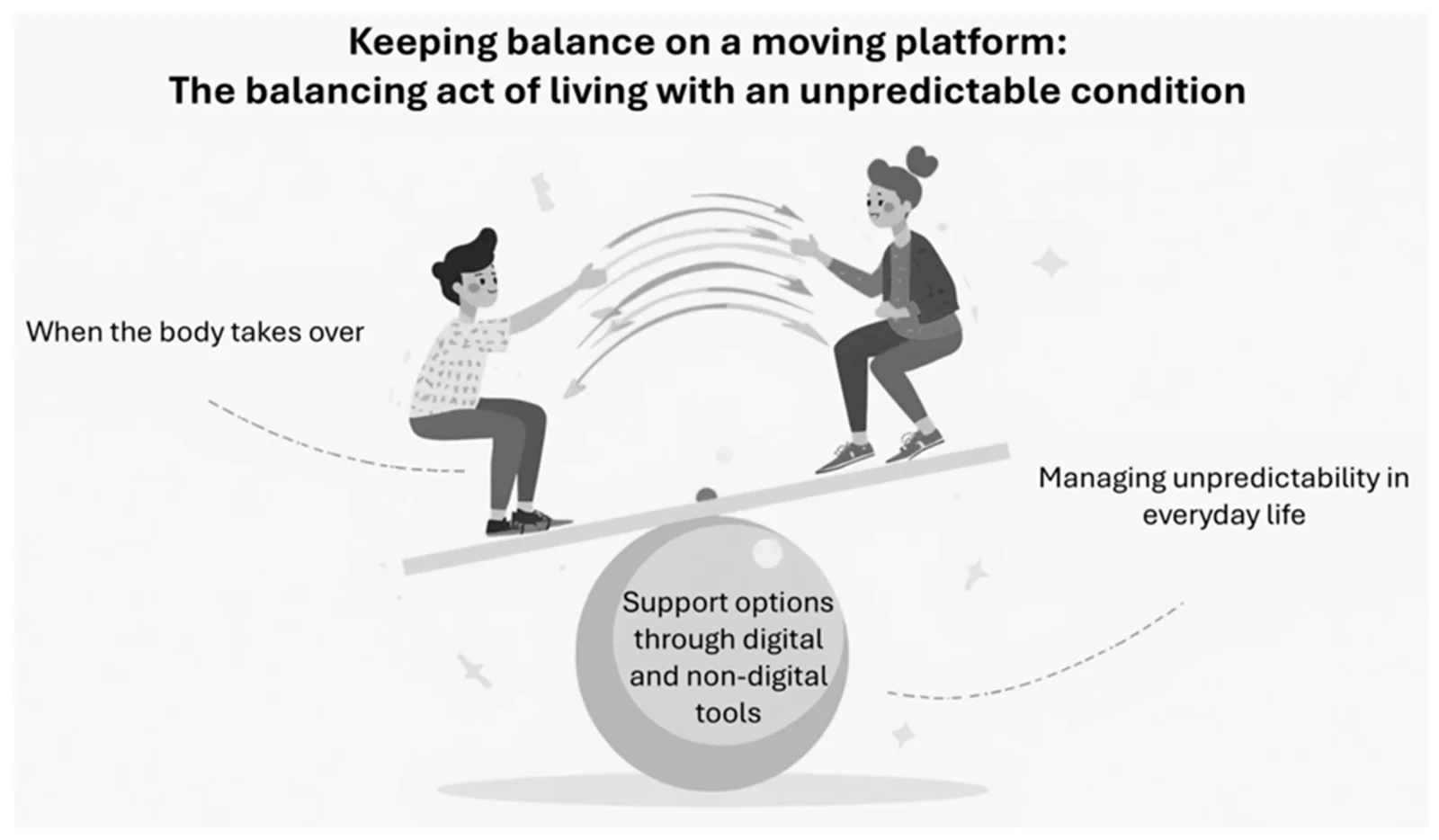
Keeping balance on a moving platform: the balancing act of living with an unpredictable condition and its three interconnected subthemes.

This balancing act was reflected across three interconnected themes: (I) When the body takes over, which captures how participants described moments when seizures or bodily changes disrupted their sense of control; (II) Managing unpredictability in everyday life, which shows how young adults managed unpredictability through routines and structure; and (III) Digital tools and online interconnection, which demonstrates how participants used or rejected digital resources to regain control, support memory, structure daily routines, or connect with others. The development of themes followed an iterative analytic process, illustrated in Figure 1, and selected quotes are presented in the text.

### 3.2. Theme I: When the Body Takes over

Many participants describe how epilepsy shapes their opportunities, relationships, and self-perception, not only through seizures but also through fear of their occurrence and concerns about others’ reactions. Across interviews, epileptic seizures were portrayed as episodes that disrupted everyday continuity, altered bodily control, and challenged participants’ sense of stability in social and relational contexts. Participants described how seizures interrupted the expected flow of daily life. Bodily sensations before, during, and after episodes created uncertainty about what would happen next. The body was often described as unpredictable and difficult to trust. In this way, epilepsy extended beyond isolated seizure events and influenced how participants understood their bodies, capabilities, and participation in everyday life.

“*People think epilepsy is just seizures… but mentally it’s exhausting to be reminded that I’m ill all the time, even though I feel capable of so many things.*”

(P4)

The diagnosis created a life circumstance where constant awareness of the body’s signals became necessary. Participants described epilepsy not only as a biomedical condition but as something that affected sensory awareness, emotional responses, and social interactions. It was often characterized as ‘constantly present’, shaping both everyday decisions and future planning.

Before seizures, many participants described becoming acutely attentive to bodily sensations. Subtle signs such as dizziness, unreality, or inner restlessness were interpreted as possible warnings, sometimes recognized by the body before they were fully cognitively processed. These early signals were experienced with ambivalence: for some, they offered a brief opportunity to prepare, whereas for others they intensified feelings of vulnerability and loss of control. For many participants, seizures were experienced as a gradual internal shift, giving rise to a sense that the body registered the impending event before conscious awareness. In these moments, time appeared to slow, as if the body were preparing for a forthcoming loss of control. However, not all participants experienced such warnings; for some, seizures occurred abruptly, disrupting the flow of everyday life without warning. Within this context, the body was often described as a messenger, even though an ambiguous one, leaving participants suspended between awareness and uncertainty:

“*I feel lucky in a way because my body warns me before a seizure. It starts with small tics in my arms and legs, as if my body is saying stop. That makes me nervous, because I know what is coming. The tics build up and create a cycle I have learned to manage a bit better, but it is frightening to feel the seizure approaching.*”

(P3)

During seizures, participants experienced a profound loss of bodily control, although this was expressed in different ways. Some described strong convulsions or collapsing movements that completely took over, leaving no sense of control. Others experienced quieter changes, such as stiffness, a fixed gaze, or small repetitive movements, while their awareness faded. For some, consciousness disappeared entirely, and they later woke up confused and dependent on others to understand what had happened. For others, some awareness remained. They could sense their body shaking or becoming still but were unable to control it, as if observing their own body without being able to act. Across participants, seizures were described as disorienting experiences that disrupted the usual connection between body and self. Whether awareness was lost or partly preserved, the body was experienced as acting independently and unpredictably.

“*I just feel like my brain and body are separated, and suddenly, in those moments, they no longer belong together… It’s like my body just goes completely into a coma.*”

(P9)

“*… It feels as though my head and body are not connected*”

(P2)

Following a seizure, participants described profound exhaustion that affected both body and mind. The body felt as heavy, sore, and depleted as if it had undergone an extreme physical strain. Many needed long periods of rest or sleep, and some experienced dizziness, muscular pain, or a lingering sense of unreality while attempting to regain orientation. For several participants, the seizure itself was lost to memory, leaving them dependent on others’ accounts and struggling to feel fully present in their own bodies. This recovery phase could last from hours to days, and was often characterized by slow thinking, reduced concentration, and a need to withdraw from everyday demands. One participant described it as follows:

“*It’s like I’ve been exercising for 24 h straight, and the exhaustion in my head is overwhelming. After my last seizure, my body felt completely wrecked, and even simple tasks were too much. Although I felt physically better the next day, it could take several days to recover mentally. There’s a lot of shame and anxiety, even though I can’t control it.*”

(P12)

Even in the absence of seizures, participants described living with a constant attentiveness to bodily signs, regulating rest, sleep, stress, and daily rhythms to prevent another episode. This ongoing alertness added to their fatigue and shaped a continuous sense of vulnerability. The emotional post-seizure experiences often included confusion, shame, anxiety, or worries about how they had appeared to others, especially when memory was absent. Across participant experiences, epilepsy emerged as more than an episodic event, affecting how participants understood their bodies and themselves in everyday life. This interpretation was reflected in participants’ descriptions of epilepsy as a broader life condition rather than solely a medical diagnosis:

“*It’s, in many ways, a different way of life. It’s like I’m living life on different terms” (P11) … “I think I experience it more as fear than as illness or seizures.*”

(P9).

### 3.3. Theme II: Managing Unpredictability in Everyday Life

Participants described how living with epilepsy shaped everyday routines, decisions, and relationships through an ongoing awareness of bodily uncertainty. The body, once taken for granted, became a tentative guide, as sensations were continuously interpreted in relation to seizure risk. Participants also described how epilepsy imposed practical restrictions that were experienced as limiting and as conflicting with their development of independence during young adulthood.

“*If I don’t get enough sleep, I know something will happen. I just don’t know what.*”

(P2).

“*I had just turned 18 when I was diagnosed, and I was in my final year of high school. It was supposed to be the time when I was becoming an adult, when no one should be telling me what I can or cannot do. Then suddenly, I was told that I couldn’t bathe alone, I couldn’t work at heights, and that I shouldn’t drink much [alcohol]. I found those restrictions difficult.*”

(P10).

“*I’m not allowed to drive, so I can’t move too far into the countryside, as it would make it difficult to get to work.*”

(P5)

Participants’ descriptions suggested that bodily sensations became a persistent focus of attention in everyday life because they could signal an approaching seizure. This ongoing alertness affected how participants approached daily activities, relationships, and future plans. One participant described how ordinary sensations, such as a ‘rumbling in my stomach’, immediately triggered concern about an approaching seizure. The interplay between body and mind was described as exhausting, not because seizures were frequent, but because the possibility of them was always near. This sense of unpredictability also shaped how participants experienced themselves in relation to others. Although they appeared ‘normal’ to others, many felt different on the inside.

“*You’re not like other people. There are many things you are not allowed to do. … I feel different.*”

(P1)

Epilepsy was often described as largely invisible yet consistently present in social contexts. Even when participating, participants described an underlying awareness of conditional safety. For some, going out, staying up late, or being away from familiar people was often overshadowed by the question of what might happen. Epilepsy also reshaped participants’ sense of time, structuring daily routines, requiring rest, and introducing uncertainty about the future. Anticipation of seizure recurrence influenced decisions related to education, work, and family life.

“*Fortunately, my epilepsy is not severe. I don’t have daily seizures because I take my medication and follow the necessary precautions. One of my biggest fears is having a seizure while holding my child. During pregnancy, we received a lot of safety guidance. There are many things you are not allowed to do.*”

(P8)

For others, however, epilepsy was experienced as more severe and disruptive:

“*Even though I didn’t have seizures all the time, I still felt a bit different, also because a lot of epilepsy medication makes you gain weight. It affects every aspect of your life.*”

(P5)

Cognitive difficulties deepened this sense of disruption. Memory lapses, difficulty concentrating, and challenges with planning all influenced how participants approached everyday tasks. One participant described the cognitive changes as ‘the biggest problem’, affecting her ability to keep up at school and trust her own capacities. These difficulties were experienced both as invisible and intrusive, subtly reshaping how participants saw themselves and what they believed they could do. In response to ongoing uncertainty, participants described developing practical strategies to create structure and reduce perceived risk, such as prioritizing sleep, adhering to medication routines, pacing activities, and engaging in self-care.

“*It has caused cognitive difficulties, which affect me most in everyday life. Because they are invisible, they are also hard for others to understand. I struggle with attention, concentration, memory, and I find it difficult to structure my day. My sense of time is impaired, and I need help planning what is realistic*”

(P7)

“*I try to train my brain as much as possible, because I know the effect of seizures and it doesn’t make it better.*”

(P5)

For many, these adjustments became integrated into daily routines. Yet, even with these strategies, unpredictability remained a defining characteristic. It influenced mood, decisions, and social participation. Across participants’ experiences, we interpreted everyday life with epilepsy as involving a continuous negotiation between independence and caution, between feeling ordinary and feeling fragile, between trusting the body and doubting it. Managing unpredictability was portrayed as an ongoing task of living with epilepsy in young adulthood, requiring continuous adjustment and adaptation.

### 3.4. Theme III: Support Options Through Digital and Non-Digital Tools

Participants described using both digital and non-digital tools as strategies to manage everyday uncertainty related to epilepsy. Digital tools included smartphone diaries, seizure-tracking apps, and timers, which were used to record seizures, support medication management, and compensate for memory difficulties. For participants experiencing cognitive difficulties, these tools could reduce the cognitive demands of remembering and organizing information.

“*I keep a diary of it on my phone because my memory is terrible.*”

(P2)

Other participants preferred non-digital strategies, including physical calendars, visual planning boards, handwritten notes, and established daily routines. Participants emphasized the importance of having information visibly available and physically accessible. These strategies supported planning and everyday structure and were described as fitting better with some participants’ individual preferences and needs.

“*I have a big board where I create a weekly schedule. It’s to make it visual and physical because it’s easier to relate to*”

(P1)

Online communities likewise carried different meanings depending on the young adults’ needs and sense of identity. For some, digital spaces offered emotional connection and recognition that could not be found elsewhere. Participants described these communities as providing a sense of connection and mutual support through contact with others who shared similar experiences.

“*I am part of a community where we support each other… a bond spread across the whole world.*”

(P12)

“*On difficult days, it helps to share feelings with others with epilepsy who truly understand, unlike parents. A like-minded person you can chat with; it helps a lot.*”

(P1)

Others approached these spaces from a distance, engaging mainly as observers. By reading rather than posting, they could draw reassurance and insight without exposing themselves or allowing epilepsy to become too central in how they appeared to others. This pattern was interpreted as a way of balancing connection with privacy.

“*I never post anything myself, but it is reassuring to read that others feel the same way.*”

(P10)

Finally, some young adults chose to avoid online communities entirely. For them, illness-focused online communities could make epilepsy feel too central to who they were. Stepping back from these groups was a way of maintaining a sense of normality and not letting epilepsy define their identity.

“*I can still function in everyday life, so I don’t think being part of groups centered around epilepsy would help me.*”

(P8)

Overall, tool use varied according to participants’ cognitive needs, everyday routines, and personal preferences. Some used digital tools to compensate for memory and organizational difficulties, whereas others preferred non-digital strategies that provided structure without increasing the focus on epilepsy. No clear pattern in tool use was identified according to reported seizure burden. However, participants’ experiences suggested that cognitive difficulties may have played a role in how some perceived and used support tools, although this was not consistent across participants. Engagement with online communities similarly ranged from active participation to passive observation or deliberate non-use, reflecting different preferences for support, privacy, and the role of epilepsy in everyday life.

## 4. Discussion

This study explored how young adults experience and negotiated everyday life with epilepsy. The findings revealed a central balancing act, captured in the main theme “*Keeping balance on a moving platform*”, reflecting participants’ continuous efforts to maintain stability while responding to the unpredictability of the condition. Seizures, cognitive difficulties, and concerns about safety and social exposure shaped how participants organized daily routines, interpreted bodily signals, and made decisions in personal and social contexts. Their strategies to maintain stability were practical and adaptive, highlighting the complexity of self-management during a life stage characterized by expanding autonomy, identity formation, and increasing responsibility. These findings highlight the importance of nursing care that supports young adults in balancing independence with the ongoing demands of living with epilepsy.

The findings suggest that unpredictability is not limited to seizure events but constitutes a pervasive condition that shapes how young adults relate to their bodies, anticipate risks, and engage in everyday life. While previous research has identified unpredictability as a defining challenge in epilepsy [6], the present study extends this understanding by demonstrating how unpredictability is continuously managed and negotiated in daily life. For young adults in particular, this ongoing negotiation intersects with major developmental transitions related to education, employment, relationships, and identity formation [2,3,10,16]. In this context, epilepsy not only affects health but also influences how independence is achieved and sustained. For neurological nurses, this emphasizes the importance of addressing developmental transitions alongside symptom management during clinical encounters.

Managing epilepsy was experienced not only as a practical task but as an ongoing cognitive and emotional effort. Participants described continuous monitoring of bodily sensations, anticipation of potential seizures, and adjustment of routines, reflecting a form of self-management that extends beyond traditional behavioral strategies [10,14,16]. Cognitive difficulties were a prominent concern and affected both functional capacity and emotional well-being, consistent with previous research [13,18,28]. The present study adds nuance by showing how young adults actively compensate for these challenges through personalized strategies that also help maintain a sense of control in an otherwise unpredictable condition. This suggests that nursing support should extend beyond seizure management to include cognitive strategies, emotional support, and individualized guidance that reflects the realities of everyday life.

The use of digital and non-digital tools reflects different ways of managing everyday uncertainty while preserving a sense of normality. Importantly, these tools are understood here not as interventions in themselves, but as part of how young adults negotiate everyday life with an unpredictable condition. Participants primarily drew on technologies already embedded in their everyday lives, such as smartphones, calendars, reminders, and note-taking tools, to support memory, structure routines, and reduce cognitive burden. Their value appeared to lie less in being health-specific solutions and more in being familiar, accessible, and easy to incorporate into everyday life. These findings suggest that meaningful digital support does not necessarily require the development of new, specialized health technologies. Instead, there may be value in recognizing and building upon the tools young adults already use in their everyday practices. By relying on familiar technologies, participants were able to manage aspects of their condition while maintaining a sense of normality and avoiding a situation in which epilepsy became overly dominant in how they understood themselves or were perceived by others. From this perspective, digital support appears less as a matter of technological innovation and more as a question of alignment with everyday practices, preferences, and identity. For nurses, this highlights the importance of exploring how young adults already use familiar technologies in their daily lives and incorporating these strategies into individualized self-management support rather than assuming that disease-specific digital interventions are always required.

Participants adopted or rejected tools not only for practical utility but also on how well they aligned with their identity and everyday life. This variation supports previous research showing that engagement with health-supporting tools depends on personal preferences and perceived relevance [29,30]. In the present study, decisions about using technology were also shaped by a desire to balance support needs with maintaining a sense of normality, highlighting how self-management may therefore be understood not only as symptom control, but as closely intertwined with identity work and social participation. At the same time, reliance on general-purpose technologies may not meet all support needs, particularly for those with more complex symptoms or higher levels of cognitive burden. Future interventions may therefore benefit from combining clinical relevance with flexibility and sensitivity to how young adults wish to live their everyday lives. While previous research has described the psychosocial and developmental challenges of epilepsy and the complexities of managing the condition alongside key developmental transitions in young adulthood [2,3,8,11,28], the present study extends this knowledge by showing how unpredictability, cognitive demands, identity, and independence are experienced as interconnected and continuously negotiated in everyday life. Recognizing these interconnected challenges may help nurses provide more individualized, developmentally appropriate, and person-centered support for young adults living with epilepsy.

### 4.1. Implications for Research and Practice

This study has several implications for neurological nursing practice. Clinically, the findings underline the importance of person-centered approaches that support self-management as an ongoing cognitive and emotional process, including strategies for managing cognitive difficulties, structuring daily routines, and navigating autonomy within necessary restrictions. In clinical practice, nurses may explore how young adults experience and respond to bodily warning signs, alongside less visible aspects of everyday cognitive functioning, including memory, concentration, time management, and the cognitive effort required to maintain everyday routines. Conversations may also address how young adults balance independence and safety and identify the digital or non-digital strategies they already use to support everyday functioning. Neurological nurses have a key role in supporting young adults through transitions into education, employment, and family life. This support should be individualized and include person-centered, transition-focused care developed in collaboration with young people [2,16,31].

The present study suggests that future interventions should address the impact of unpredictability on daily functioning within the developmental context of young adulthood. This perspective complements previous research highlighting the importance of transition-focused care [2,16,31]. Future research should also explore how digital and non-digital tools can support autonomy while preserving a sense of normality, with attention to how interventions are designed, for whom, and with what content. Approaches should be tailored to cognitive functioning and developed in collaboration with young adults to ensure relevance and usability [29,30,31]. Future intervention studies should evaluate whether nursing interventions tailored to developmental stage, cognitive functioning, and everyday self-management improve participation, well-being, and long-term self-management outcomes.

### 4.2. Strengths and Limitations

A strength of this study is its in-depth exploration of everyday experiences among young adults with epilepsy, a group that remains underrepresented in qualitative research. This specific focus allowed experiences related to independence and developmental transitions to be explored within the context of young adulthood. The use of individual interviews allowed for rich, detailed accounts of how participants understood and managed living with an unpredictable condition. Furthermore, the inclusion of participants from different regions and living situations contributed to variation in perspectives, enhancing the information power of the sample [23]. The sample size was considered sufficient based on the concept of information power [23]. The aim and specific sample of young adults with lived experience of epilepsy provided data closely aligned with the phenomenon under study. The individual interviews facilitated in-depth dialogue, with probing and clarifying questions allowing participants to elaborate on their experiences and generating rich and detailed data. The hermeneutically informed reflexive thematic analysis was chosen to enable an in-depth and iterative interpretation of patterns of meaning within and across participants’ experiences [19,20]. Thus, the specificity of the sample, the focused aim, the depth and quality of the interview dialogue, and the interpretative analytical strategy increased the information power of the data and supported the adequacy of 12 interviews [23]. In addition, the analysis was informed by ongoing reflexive engagement with the data, acknowledging the active role of the researchers in interpreting participants’ experiences [19].

However, several limitations should be considered. Participation was voluntary, which may have favored individuals who were more comfortable discussing their condition. Recruitment through the Danish Epilepsy Association and social media may also have favored individuals engaged with patient organizations or online platforms or with greater digital access. Young adults with limited digital access, lower engagement with patient organizations, or less interest in online epilepsy-related communities may therefore be underrepresented. The sample was predominantly female, with 11 of the 12 participants being women, which may have limited the representation of experiences among young men with epilepsy. These sampling characteristics should be considered when interpreting the diversity and transferability of the findings. Moreover, the Danish healthcare and social context, as well as variation in participants’ employment situations, should also be considered when assessing transferability to other settings. This should be considered when applying the findings to nursing practice in healthcare systems or cultural contexts that differ from the Danish setting. In addition, the use of online interviews, while practical and increasing accessibility, may differ from in-person interviews in ways that influence rapport and depth of disclosure [24]. Finally, as with qualitative research more broadly, the findings are contextually situated and should be interpreted as analytical insights rather than generalizable outcomes [19]. Nevertheless, the findings provide transferable insights that may inform person-centered nursing care for young adults living with epilepsy. Whether these insights may also be relevant to young adults living with other neurological conditions warrants further investigation.

## 5. Conclusions

This study shows that living with epilepsy in young adulthood involves an ongoing effort to maintain balance in the context of unpredictability, captured in the main theme “*keeping balance on a moving platform.”* Epilepsy was experienced not only as episodic seizures but as a pervasive condition shaping bodily awareness, daily routines, and social participation. Young adults continuously negotiate between independence and vulnerability while managing cognitive challenges and uncertainty in everyday life. Nursing support may be strengthened by building familiar tools and strategies that young adults already use in their everyday lives. For neurological nurses, the key implication is to look beyond seizure control by exploring cognitive challenges, everyday self-management, and the balance between independence and safety together with young adults.

## Figures and Tables

**Table 1:** Participant Demographics.

Participant (P#)	Age *	Sex	Marital Status	Employment	Diagnostic Evaluation/Treatment Follow-Up	Epilepsy Duration *	Seizure (Frequency, Last Seizure)	Seizures with or Without Impaired Awareness (+/−)	Comorbidities	Self-Reported Cognitive Problems
P1	18	F	Living with parents	Student	Specialized epilepsy hospital/Neurology outpatient clinic	14	Many, two years ago	Both	-	Concentration
P2	25	F	Living with a partner	On sick leave	Specialized epilepsy hospital/Neurology outpatient clinic	13	Six seizures per year, period free of seizures, six months ago	Both (most +)	Neurological disorder, Chronic back pain	Concentration, learning & memory
P3	22	M	Living with a partner	Student	Specialized epilepsy hospital/General practitioner	4	2–3 years ago	Both	-	Memory
P4	20	F	Living with parents	Student	Neurology outpatient clinic	16	Three weeks ago	Both	-	-
P5	26	F	Living with a partner	Employed	Specialized epilepsy hospital/Neurology outpatient clinic	16	1–2 seizures per month, period free of seizures	Both	-	-
P6	18	F	Living with parents	Student	Neurology outpatient clinic	1	Eight months ago	Both	Stress-related disorder	Concentration, learning & memory
P7	27	F	Living with parents	On sick leave	Neurology outpatient clinic	7	Twice monthly (+), 3–4 weekly (−), last week	Both (most +)	Systemic lupus erythematosus, severe allergy	Concentration, learning & memory, + executive function, and sense of time.
P8	26	F	Living with partner, 2 children	Employed/maternity leave	Neurology outpatient clinic	13	Period free of seizures, a few days ago	Both	Migraine	Concentration & memory
P9	27	F	Living with a partner, 1 child	Employed	Neurology outpatient clinic	9	Period free of seizures, two months ago	Both (Most −)	-	Concentration & memory
P10	21	F	Living with parents	Employed	Neurology outpatient clinic/general practitioner	3	Twice weekly	Both (Most +)	Depression	Concentration & memory
P11	29	F	Living with a partner, 2 children	Employed/maternity leave	Neurology department/Neurology outpatient clinic	17	Daily pre-seizure symptoms, Last seizure >10 years ago	Both (Most −)	-	-
P12	26	F	Living with a partner	Employed	Neurology department/Neurology outpatient clinic	10	One weekly (−), last week (+)	Both	-	Memory

* years, (+/−) = with or without impaired awareness.

**Table 2:** The interview guide.

Topics	Examples of Questions
Opening	Introducing the aim and explaining ethical rights. Start by telling me a little about yourself
Bodily experiences	How do you experience your condition? How does your treatment affect you?
Social relationships	How does your illness influence your relationships?
Everyday environments	How do you experience everyday life with your condition? —at home, outside, or online?
Supportive technologies	How do you view your treatment and support? What kind of technology do you use?
Experiences over time	How has your experience of the condition changed over time? How does it affect your thoughts about the future?
Ending	Anything you would like to add?

**Table 3:** Example of the analytical process.

Interview Questions	Selected Quotes (Examples)	Sub-Themes	Theme	Main Theme
How do you experience your illness?	“*I experience it primarily as a mental burden, because many people think of epilepsy as just convulsive seizures, and in daily life you may walk around feeling fine, while in reality it is much more than that*” (P5)	Epilepsy is not just a diagnosis and shapes life as a condition	When the body takes over	Keeping balance on a moving platform
	“*It’s kind of a scary thing to carry around with you, I think*” (P2)			
How do you experience your everyday life with your condition?	“*It went on for about a year, and it happened once a minute. So, I would be gone for 30 s, then come back not knowing where I was, and then it would start again. I was basically out of it for a whole year*” (P1)	Epilepsy and seizures shape bodily and self-experiences		
	“*I often forget what happened, and the memory comes back slowly. When I wake up, my whole body feels beaten up, like after the hardest workout, because everything has cramped. I’m exhausted, sore, my head hurts, and I wake up on the floor thinking, okay, now I know what just happened*” (P2)			
How does it affect your thoughts about the future?	*“I don’t even feel like I think about it.… It has almost just become part of everyday life”* (P9)	Epilepsy shapes life perspective		
How do you experience your everyday life with your condition?	“*I am very conscious of my sleep. I have struggled with sleep problems and anxiety related to sleep, particularly throughout last year. It still affects me a little, and I am very much about control. I am very conscious of getting enough sleep because I know what consequences it can have for me”* (P5)	Recognizing and managing seizure triggers	Managing unpredictability in everyday life	
	*“Since I started running, going to bed earlier, doing breathing exercises, and leaving my phone downstairs before going to sleep, it has also helped with my epilepsy. I need between 9 and 10 h of sleep for things to work in relation to my epilepsy and my seizures”* (P3)			
	*“It’s in my mind all the time, that thing about, you know, actively taking care of myself”* (P5)	Living within restrictions		
	*“At that time, when I was diagnosed, my everyday life was built around my epilepsy. My daily routines were changed to accommodate my epilepsy”* (P8)			
	*“In my everyday life, I don’t feel I’m limited. Of course, there are some things I don’t want to do because of my epilepsy. I can be a little nervous about what might happen, but I think that’s quite normal”* (P3)			
How do you experience your everyday life with your condition?	*“I have planned, I have a schedule, a whiteboard where I write…”* (P6)	Creating structure and routines		
	*“I think it is also because my memory is so poor that I have developed such a strong need to be able to control everything and have everything written down, so things like notes and things like that”* (P12)			
	*“I’ve made it into a fixed routine, instead of having to carry medication with me everywhere. I just make sure I take it morning and evening”* (P2)			
	*“We feed our cat breakfast. And then I usually take my medication. It is a routine. Like when you go to bed, you also brush your teeth in the evening. You’ve just gotten used to it. It’s the same with the medication. I’m used to it”* (P3)			
How do you use tools or technology in everyday life?	*“I have had a timer running since I took the medication for the first time. Just to see how long the period is. It has taken some time, but it has also turned out well in the end”* (P3)	Using familiar digital and non-digital tools	Support options through digital and non-digital tools	
	*“But now I rely on having everything written down in the smallest detail. I don’t know why, but it works better for me when it’s in such a good old-fashioned calendar”* (P5)			
How do you use online communities or digital resources related to epilepsy?	*“We have entered a completely different era than 20 years ago, so instead of feeling small and sorry for myself, I am part of a community where we support each other. Where you realize that you are part of a bond spread across the whole world, where you help yourself”* (P8)	Online communities		
	*“You know what it feels like when it’s the middle of the night and it feels like no one understands you, but there is someone who does… I know what it feels like to sit there at night and feel that you have lost control of your brain and yourself”* (P12)			
	*“I have used the Epilepsy Association’s website. Sometimes I watch videos as well. One of my favorite actors, who is also a dancer, has epilepsy, and I have watched interviews with her… But mostly I have used written information”* (P4)			

## Data Availability

The data supporting the findings of this study are not publicly available due to the sensitive nature of the interview data and the risk of participant identification but may be available from the corresponding author upon reasonable request, subject to applicable data protection regulations.

## Public Involvement Statement

The Danish Epilepsy Association assisted with participant recruitment by distributing study information through its communication channels. Patients or members of the public were not involved in the design, conduct, analysis, interpretation, or dissemination of the research.

## Guidelines and Standards Statement

This manuscript was prepared in accordance with the Standards for Reporting Qualitative Research (SRQR) [23] and the author guidelines of *Nursing Reports*.

## Use of Artificial Intelligence

Microsoft 365 Copilot (Microsoft Corporation, Redmond, WA, USA) was used to support language editing and grammar during manuscript preparation, and Canva (Magic Design; Canva Pty Ltd., Sydney, Australia. Web-based graphic design platform accessed the 1st of June 2026) was used to support layout in Figure 1. No generative AI or AI-assisted tools were used to generate study data, conduct the analysis, or develop interpretations. All AI-assisted content was reviewed and verified by the authors, who take full responsibility for the final content.
